# Establishment of a reverse transcription recombinase-aided amplification detection method for porcine group a rotavirus

**DOI:** 10.3389/fvets.2022.954657

**Published:** 2022-09-15

**Authors:** Yushun Wang, Mincai Nie, Huidan Deng, Siyuan Lai, Yuancheng Zhou, Xiangan Sun, Ling Zhu, Zhiwen Xu

**Affiliations:** ^1^College of Veterinary Medicine, Sichuan Agricultural University, Chengdu, China; ^2^Animal Breeding and Genetics Key Laboratory of Sichuan Province, Sichuan Animal Science Academy, Chengdu, China; ^3^Livestock and Poultry Biological Products Key Laboratory of Sichuan Province, Sichuan Animal Science Academy, Chengdu, China; ^4^College of Veterinary Medicine Sichuan Key Laboratory of Animal Epidemic Disease and Human Health, Sichuan Agricultural University, Chengdu, China

**Keywords:** porcine rotavirus, VP6 gene, RT-RAA, RT-qPCR methods, detection assay

## Abstract

Porcine rotavirus type A (PoRVA) is the main cause of dehydration and diarrhea in piglets, which has a great impact on the development of the pig industry worldwide. A rapid, accurate and sensitive detection method is conducive to the monitoring, control, and removal of PoRVA. In this study, a PoRVA real-time fluorescent reverse transcription recombinase-aided amplification (RT-RAA) assay was developed. Based on the PoRVA VP6 gene, specific primers and probes were designed and synthesized. The sensitivity of RT-RAA and TaqMan probe-based RT-qPCR was 7 copies per reaction and 5 copies per reaction, respectively. The sensitivity of the RT-RAA method was close to TaqMan probe-based RT-qPCR. The detection results of RT-RAA and TaqMan probe-based quantitative real-time RT-PCR methods were completely consistent in 241 clinical samples. Therefore, we successfully established a rapid and specific RT-RAA diagnostic method for PoRVA.

## Introduction

Rotavirus (RVs) is an important enteric pathogen, a zoonotic virus of the genus Rotavirus, and also a double-strand RNA virus (1). VP6 proteins are divided into 9 groups according to their antigenic relationship (A-J) (2). Group A rotavirus (GARV) is one of the main causes of viral gastroenteritis with multiple hosts, including humans, cows, pigs, horses, dogs, cats, et al. (3–5). It is estimated that about 215,000 children died from RV each year, and it is the primary cause of death among infants worldwide (6). GARV is one of the main infectious diseases with a huge impact on the porcine industry (7). RV can cause vomiting, diarrhea, dehydration, and other clinical symptoms in piglets, resulting in slow growth or even death of piglets and bringing significant economic losses to the pig industry (8). Rotavirus strains have the ability to spread across species and new genotypes may emerge in different hosts, which poses a serious threat to human and animal health. Therefore, we established a rapid and specific RT-RAA diagnostic method for PoRVA.

At present, serological and etiological tests are the main diagnostic methods of PoRVA. Serological tests major including immunochromatographic test strips and ELISA (9, 10). Etiological detection usually includes methods such as virus isolation (11), RT-PCR (12), RT-qPCR (13) and loop-mediated isothermal amplification (LAMP) (14). These methods play a very essential role in the prevention, control, and elimination of PoRVA, but it is time-consuming, complex to operate, expensive materials, and requires experienced technicians. This prevents them from being widely used in equipment-limited environments or field testing. Therefore, it is necessary to develop a rapid, simple, and reliable diagnostic method for PoRVA.

Recombinase-aided amplification (RAA) assay is a new technology capable of isothermal amplification, which does not require complicated temperature settings, but only needs to be performed under a constant temperature of 37–42°C. Three key enzymes are present in the RAA assay: a recombinase (Bind specific primers to templates); a DNA polymerase (Product expansion and extension) and a single-stranded DNA-binding protein (15–17). RT-RAA detection method can directly use RNA as a template for pathogen detection (18, 19). Amplification products can not only be detected in the laboratory by some common laboratory instruments but also can be detected in the field by using portable instruments, such as lateral flow meters (20), and portable blue light instruments (16). Up to now, it is unknown that many kinds of organisms are detected by using the RAA method for pathogenic microorganisms (16, 20, 21).

However, the RT-RAA method has not been reported in PoRVA detection. In this study, a rapid and efficient RT-RAA method for PoRVA detection was established, and make a preliminary evaluation on it.

## Methods

### Clinical samples

A total of 241 clinical samples (small intestine contents and Feces) Suspected symptoms of PoRVA infection were collected in 2021 from the porcine farms located in Sichuan province, southwestern China. Suspend intestinal contents and feces in PBS following that −80°C for storage and use.

### Primer and probe

Multiple VP6 genes of PoRVA were downloaded from the GenBank database, the most conserved sequence was obtained by the DNASTAR software (DNASTAR Inc., Madison, WI, USA) for primer and probe design. Oligonucleotide primers and probes (Shanghai Sangon Biotech Co., Ltd.) were designed based on the VP6 gene sequence for the RT-RAA assay. Oligonucleotide primers and probes for TaqMan probe-based RT-qPCR methods were derived from the TaqMan probe-based quantitative real-time RT-PCR Kit (Shanghai Zeye Co., Ltd.) (Supplementary Tables 1, 2).

### RNA extraction

After nucleic acid extraction of the sample using a Virus RNA/DNA Extraction Kit (Hong Kong Kefit Co., Ltd.), −80°C is stored until use.

### Production of standard control

Two recombinant plasmids for RT-RAA and TaqMan probe-based RT-qPCR were constructed, respectively. And then the sequence correctness of the two recombinant plasmids was verified by Sanger sequencing (Shanghai Sangon Biotech Co., Ltd.).

### Primer selection and real-time RT-RAA assay

This study was performed using a fluorescent RT-RAA nucleic acid amplification kit (Jiangsu Qitian Gene Biotechnology Co., Ltd.). The total reaction system of RT-RAA is shown in the Supplementary Table 3. After the system configuration was completed, it was quickly transferred to a fluorescence quantitative PCR instrument at 39°C. The constant temperature was 39°C, and each cycle was 1 min, 30 cycles. Use nucleic acid-free water for negative control.

Pair the 3 forward and 3 reverse primers of RT-RAA with each other. Primers 1 to 9 were R-F1-R1, R-F1-R2, R-F1-R3, R-F2-R1, R-F2-R2, R-F2-R3, R-F3-R1, R-F3-R2, and R-F3-R3, and RT-PCR detection was performed using the above primer combinations. After recycling the target strip, it is connected with the pMD-T vector.

And the ligated vector was sequenced at Sangon Bioengineering Co., Ltd. (Shanghai, China). The sequencing results were verified to be PoRVA sequences. The PoRVA vectors with correct sequencing results were verified using 9 pairs of combinations, and the primers were selected by the intensity of the fluorescence value and the peak time of fluorescence.

### Sensitivity and specificity of real-time RT-RAA

Serial dilutions of the RT-RAA recombinant plasmid ranging from 10^5^ to 10^−1^ copies per reaction were used to evaluate the sensitivity of the real RT-RAA assay. Serial dilutions of the TaqMan probe-based RT-qPCR recombinant plasmid ranging from 10^7^ to 10^−1^ copies per reaction were used to evaluate the sensitivity of TaqMan probe-based RT-qPCR. To evaluate the specificity of the RT-RAA assay by the detection of positive clinical samples such as Porcine reproductive and respiratory syndrome (PRRSV), Japanese encephalitis virus (JEV), Atypical porcine pestivirus (APPV), Seneca Valley virus (SVV), Classical swine fever virus (CSFV) and Getah virus (GETV).

### Clinical samples analysis

To assess clinical effects, 241 clinical samples were detected by real-time RT-RAA and TaqMan probe-based quantitative real-time RT-PCR Kit (Shanghai Zeye Co., Ltd.), and check the match rate of the two methods. The TaqMan probe-based RT-qPCR system and process are shown in Supplementary Table 4.

### Statistical analysis

The detection limits of real-time RT-RAA and TaqMan probe-based RT-qPCR were analyzed by Probit regression at 95% CI using the IBM SPSS software. Real-time RT-RAA and TaqMan probe-based RT-qPCR results were determined using kappa and *p*-values.

The experimental procedure was approved by the Institutional Animal Care and Ethics Committee of Sichuan Agricultural University. The “Guidelines for Experimental procedure” of the Ministry of Science and Technology (Beijing, China) were followed.

## Results

### Results of best primer screening

**Figure 1:**
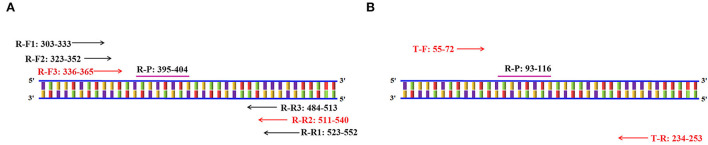
Schematic diagram of primer amplification. **(A)** Schematic diagram of the RT-RAA primer screening. The numbers in the primer name represent the position of the oligonucleotides in the conservative region of the VP6 gene. **(B)** Schematic diagram of Taqman probe-based RT-qPCR primers amplification. The numbers in the primer name represent the position of the oligonucleotides in the conservative region of the VP6 gene.

**Figure 2:**
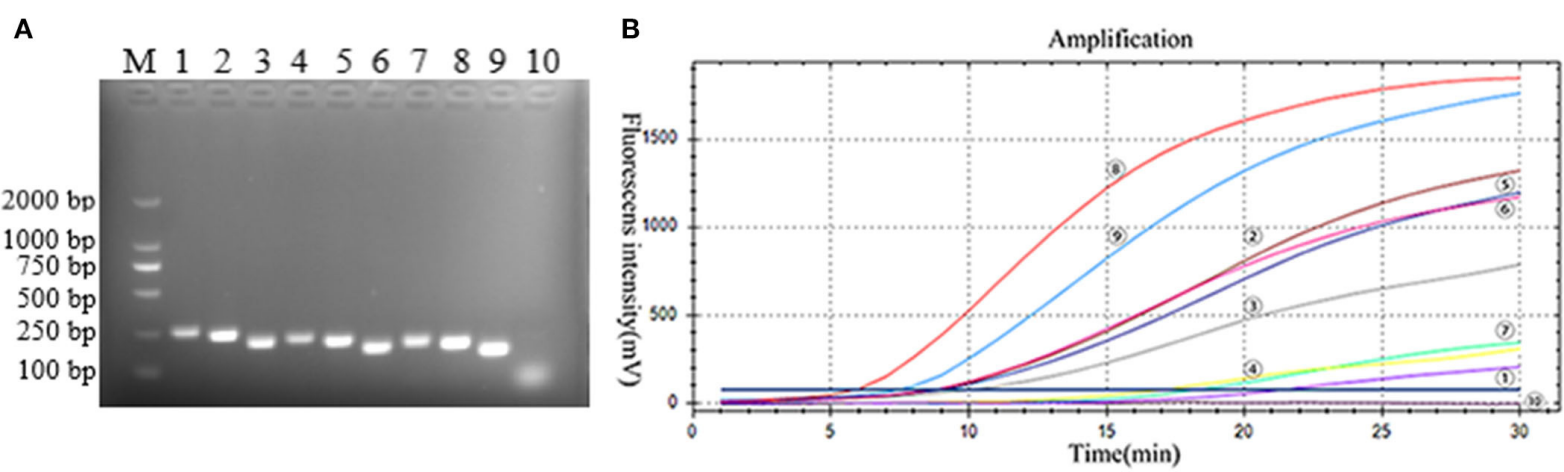
**(A)** Best primer screen. M: DL2000 marker; 1–9: 9 pairs of PoRVA primers, 1: R-F1-R1, 2: R-F1-R2, 3: R-F1-R3, 4: R-F2-R1, 5: R-F2-R2, 6: R-F2-R3, 7: R-F3-R1, 8: R-F3-R2, 9: R-F3-R3; 10: Negative Control. **(B)** RT-RAA primer screening results. :R-F1-R1, : R-F1-R2, : R-F1-R3, : R-F2-R1, : R-F2-R2, : R-F2-R3, : R-F3-R1, : R-F3-R2, : R-F3-R3; : negative control. TT value: : 6.16, : 7.84, : 9.19, : 9.34, : 9.27, : 10.80, :17.72, : 18.96, : 20.20, : none.

The primers and probes of the RT-RAA and Taqman probe-based RT-qPCR were used to detect the PoRVA in this study (Figure 1). RT-RAA primers were screened with 1 × 10^4^ copy number recombinant plasmid. PoRVA was able to be successfully amplified specific bands by RT-PCR with these nine pairs of primers (Figure 2A), and the sequencing results are consistent with the PoRVA sequence. Of all the primers, the fluorescence value is the highest, and the earliest peak appears in the reaction system of R-F3 and R-R2 (Figure 2B). Therefore, in subsequent experiments, R-F3 and R-R2 were combined as primers for RT-RAA.

### Results of sensitivity analysis

**Figure 3:**
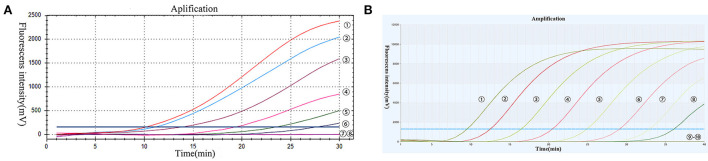
**(A)** The results of RT-RAA sensitivity analysis: RT-RAA PoRVA plasmid as standard, -: 1 × 10^5^-1 × 10^−1^ copies per reaction sequentially; : negative control. **(B)** The results of TaqMan probe-based RT-qPCR sensitivity analysis: TaqMan probe-based RT-qPCR PoRVA plasmid as standard, -: 1 × 10^7^-1 × 1 0^−1^ copies per reaction sequentially; : negative control.

The PoRVA sensitivity was measured after Serial dilution of the RT-RAA and TaqMan probe-based RT-qPCR recombinant plasmid from 105 to 10–1 copies per reaction and 107–10–1 copies per reaction, respectively. Eight tests were performed for each dilution to evaluate the repeatability of the assay (Supplementary Tables 5, 6). The minimum detection limit of real-time RT-RAA assay was 7 copies per reaction and the minimum detection limit of TaqMan probe-based RT-qPCR methods was 5 copies per reaction. As shown in Figure 3, the higher the recombinant plasmid concentration, the earlier the reaction peak.

### Results of specificity analysis

**Figure 4:**
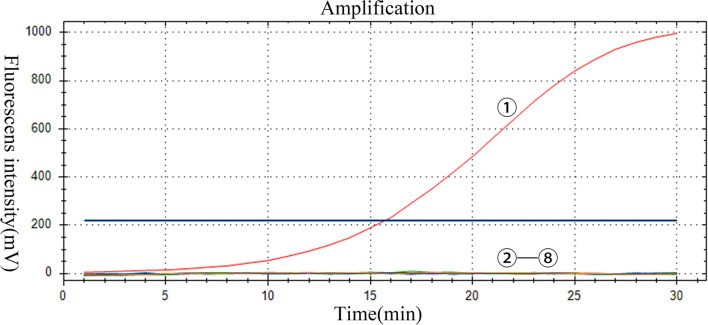
Specificity of the RT-RAA assay for PoRVA. -: PoRVA, PRRSV, JEV, APPV, SVV, CSFV, and GETV virus RNA, respectively; : negative control.

The specificity of the assay was evaluated by using positive samples containing other RNA viruses. Only PoRVA sample results are positive. However, the PoRVA positive samples for other viruses and all negative control samples were negative in real-time RT-RAA detection (Figure 4). Therefore, this method proved to have high specificity in the detection of PoRVA. These results demonstrate that the specificity of the real-time RT-RAA assay was 100%.

### Results of clinical samples were used to evaluate PoRVA real-time RT-RAA assay

To assess clinical effects, 241 clinical samples were detected by real-time RT-RAA and TaqMan probe-based RT-qPCR (Supplementary Table 7) shows the comparison of RT-RAA and TaqMan probe-based RT-qPCR detection results. PoRVA RT-RAA assay has a kappa value of 1 (*p* < 0.001), and 17 positive samples were successfully detected from 241 samples by the RT-RAA method, in full agreement with TaqMan probe-based RT-qPCR results.

## Discussion

Porcine rotavirus type A is an important pathogen that causes viral diarrhea, and leads to dehydration diarrhea in piglets, posing a considerable threat to China's pig industry (11, 22, 23). Many published studies have proved rotavirus can be transmitted between porcine and humans in China. PoRVA evolves in its molecular epidemiology with time, this has led to the ineffectiveness of conventionally developed vaccines. To monitor PoRVA continuously and effectively over time, several diagnostic methods have been developed. These methods play a very essential role in the prevention, control, and elimination of PoRVA infection. However, these methods are time-consuming, complex to operate, expensive materials, and require experienced technicians. Therefore, it is necessary to develop a rapid, simple, and reliable diagnostic method for PoRVA.

In this study, a new real-time fluorescence RT-RAA method for PoRVA detection was successfully established. Under the constant temperature rapid amplification at 39°C, the fluorescent signal can be detected within 15 min, and get the result within 30 min, shorter than the PoRVA RT-qPCR method established by Marthaler et al. (13). The minimum detection limit of real-time RT-RAA assay was 7 copies per reaction, the sensitivity is comparable to the RT-qPCR method established by Xu and far higher than the ordinary RT-PCR (24). There was no cross-reaction of other strains in this method, which was specific. Additionally, a total of 110 clinical samples were tested to determine the clinical efficacy of the method. The real-time RT-RAA assay showed high accuracy and the match rate of RT-RAA and TaqMan probe-based RT-qPCR was 100%. The results showed that real-time fluorescence RT-RAA is a promising method for rapid, specific, and sensitive detection of PoRVA.

The RVs VP6 gene is often used to identify different subtypes of rotavirus, but in the same subtype, the VP6 gene showed a highly conserved type (25). Therefore, the VP6 is often used as the target gene for primer and probe design in PoRVA nucleic acid detection methods. In this study, the conserved sequences of PoRVA VP6 were selected to design primers and probes to ensure the high specificity of the method. At the same time, the length of the primer designed in this experiment is 35 bp, which is longer than the general primer. Thus, improving the specificity of the test. The recombinase, DNA polymerase, a single-stranded DNA-binding protein can make nucleic acid amplification at constant temperature fast in the RT-RAA reaction system. At the same time, the addition of a reverse transcription system in the RAA system can reduce the single reverse transcription step and shorten the detection time. Therefore, the PoRVA RT-RAA method can obtain results within 30 min. There are three ways to determine the results of the RAA test: Agarose gel electrophoresis, immunochromatographic test strip, and fluorescence probe method, the fluorescence probe method was more sensitive than the other two methods.

Compared with other methods, the RT-RAA method has many advantages. Firstly, the RT-RAA assay is a constant temperature nucleic acid amplification method, it can react with any thermostatic device, such as a water bath, a metal block, or even body temperature. This is especially suitable for use in rudimentary condition laboratories. Secondly, we were able to observe RT-RAA reaction products with the naked eye when we used a 480 nm blue light imager, this is practicable to the RT-RAA method in clinical rapid detection in a poorly equipped field.

In conclusion, this study established a PoRVA RT-RAA detection method with strong specificity, high sensitivity, and fast detection speed. The established PoRVA RT-RAA detection method was used in the testing of clinical samples, and the RT-RAA results were completely consistent with RT-qPCR, indicating that the testing results of RT-RAA were accurate and reliable. Therefore, in this study established RT-RAA can be utilized for the detection of PoRVA in clinical samples.

## Data availability statement

The original contributions presented in the study are included in the article/Supplementary material, further inquiries can be directed to the corresponding author/s.

## Author contributions

YW and MN designed the study. YW, MN, HD, YZ, XS, SL, LZ, and ZX conducted the experiments. YW, MN, and HD wrote the manuscript. All authors reviewed the manuscript. All authors contributed to the article and approved the submitted version.

## Funding

This study was supported by the Science and Technology Plan Project of Sichuan Province (Project No. 2020YFN0147).

## Conflict of interest

The authors declare that the research was conducted in the absence of any commercial or financial relationships that could be construed as a potential conflict of interest.

## Publisher's note

All claims expressed in this article are solely those of the authors and do not necessarily represent those of their affiliated organizations, or those of the publisher, the editors and the reviewers. Any product that may be evaluated in this article, or claim that may be made by its manufacturer, is not guaranteed or endorsed by the publisher.
