# Supplementary material for: Establishment of a reverse transcription recombinase-aided amplification detection method for porcine group a rotavirus
Source: Front Vet Sci. 2022 Sep 15;9:954657. doi: 10.3389/fvets.2022.954657 (PMC9519424; doi:10.3389/fvets.2022.954657)
Supplement: Supplementary file 1 [file Table_1.DOCX]

Table 1: Sequences of Primers and Probe for Assays for PoRVA

| Primer/Probe | Sequence (5'-3') | Length（bp） | Tm（℃） |
| --- | --- | --- | --- |
| R-F1 | CAGAGAATCACAACGAAATGGGATAGCTCCA | 31 | 68.0 |
| R-F2 | GGATAGCTCCACAATCTGAAGCATTAAGGAA | 31 | 63.3 |
| R-F3 | ATCTGAAGCATTAAGGAAATTGTCCGGCAT | 30 | 63.6 |
| R-R1 | GGCATTAATCCACATCGTCCCCATCAAATT | 30 | 63.6 |
| R-R2 | CATCGTCCCCATCAAATTATCATGCGCTGG | 30 | 66.2 |
| R-R3 | TGGCTGTGATCTATTCAGAGTAAATGATGC | 30 | 61.4 |
| R-Probe | CCGGCATTAAATTTAAGAGAATTAACTT/i6FAMdT/G/idSp//  iBHQ1dT/AATTCATCTGATA | 45 | 62.2 |
| RT-PCR-F4 | AAATGACTTTCAAACGGGAG | 20 | 52.7 |
| RT-PCR-R4 | TTTATGAAACACAAATCCGGTA | 22 | 51.3 |

Table 2: Sequences of Primers for TaqMan probe-based RT-qPCR

| Primer/Probe | Sequence (5'-3') | Length（bp） | Tm（℃） |
| --- | --- | --- | --- |
| TaqMan-F | GAGTGAAATGGCTAGAGA | 18 | 59.4 |
| TaqMan-R | GATGCTGAGTATGGAAGTA | 19 | 59.8 |
| TaqMan-Probe | FAM-CTCCACAATCTGAAGCACTGAGAA-BHQ1 | 24 | 67.7 |

Table 3: RT-RAA reaction system

| Reagent | Concentration | Content/μL |
| --- | --- | --- |
| Buffer | __ | 25.0 |
| Forward primer | 10 μM | 2.1 |
| Reverse primer | 10 μM | 2.1 |
| Probe | 10 μM | 0.6 |
| ddH_2_O and RNase inhibitor | __ | 15.2 |
| Template | __ | 2.0 |
| Reaction starter | __ | 3.0 |

Table 4: TaqMan probe-based RT-qPCR system

| TaqMan probe-based RT-qPCR system | | TaqMan probe-based RT-qPCR procedure | | |
| --- | --- | --- | --- | --- |
| Reagent | Content/μL | Temperature (℃) | Time | Cycle |
| RT-qPCR Buffer | 10 μL | 50℃ | 30min | 1 |
| RT-qPCR Enzyme mix buffer | 2 μL | 94℃ | 10min | 1 |
| RT-qPCR probe | 1 μL | 94℃ | 15s | 40 |
| ddH_2_O | 2 μL | 60℃ | 1min | 1 |
| RNA Template | 5 μL |  |  |  |

Table 5: Sensitivity of RT-RAA was detected by porcine PoRVA plasmid

| Serial dilutions of PoRVA plasmid (copies/reaction) | No. Replicates tested | No. detection | Detection rate (%) |
| --- | --- | --- | --- |
| 7×10^5^ | 8 | 8 | 100 |
| 7×10^4^ | 8 | 8 | 100 |
| 7×10^3^ | 8 | 8 | 100 |
| 7×10^2^ | 8 | 8 | 100 |
| 7×10^1^ | 8 | 8 | 100 |
| 7×10^0^ | 8 | 8 | 100 |
| 7×10^-1^ | 8 | 0 | 0 |

Table 6: Sensitivity of TaqMan probe-based RT-qPCR was detected by porcine PoRVA plasmid

| Serial dilutions of PoRVA plasmid (copies/reaction) | No. Replicates tested | No. detection | Detection rate (%) |
| --- | --- | --- | --- |
| 5×10^7^ | 8 | 8 | 100 |
| 5×10^6^ | 8 | 8 | 100 |
| 5×10^5^ | 8 | 8 | 100 |
| 5×10^4^ | 8 | 8 | 100 |
| 5×10^3^ | 8 | 8 | 100 |
| 5×10^2^ | 8 | 8 | 100 |
| 5×10^1^ | 8 | 8 | 100 |
| 5×10^0^ | 8 | 8 | 100 |
| 5×10^-1^ | 8 | 0 | 0 |

Table 7: PoRVA was detected by RT-RAA and TaqMan probe-based RT-qPCR

|  |  | RT-RAA | | Total | Kappa(κ) | p-value  of kappa |
| --- | --- | --- | --- | --- | --- | --- |
|  |  | Positive | Negative |  |  |  |
| RT-qPCR | Positive | 17 | 0 | 17 | 1 | ＜0.001 |
|  | Negative | 0 | 224 | 224 |  |  |
| Total |  | 17 | 224 | 241 |  |  |
